# Access to primary care is associated with better autoimmune hepatitis outcomes in an urban county hospital

**DOI:** 10.1186/s12876-015-0318-y

**Published:** 2015-07-28

**Authors:** Daniel Kim, Daniel Eshtiaghpour, Joel Alpern, Anuj Datta, Viktor E. Eysselein, Hal F. Yee

**Affiliations:** 1 Department of Medicine, Harbor-UCLA Medical Center, 1000 W. Carson St., Box 400, Torrance, CA 90509 USA; 2 Department of Gastroenterology, Harbor-UCLA Medical Center, Torrance, CA USA; 3 Department of Medicine, University of California, San Francisco, San Francisco, CA USA

**Keywords:** Autoimmune hepatitis, County, Safety-net, Hispanic

## Abstract

**Background:**

Autoimmune hepatitis causes chronic hepatitis and often leads to cirrhosis and death without treatment. We wanted to see if having access to primary care or insurance prior to diagnosis is associated with better outcomes for patients in an urban, public hospital with mostly socioeconomically disadvantaged Hispanic patients.

**Methods:**

We did a retrospective study at our institution. Kaplan Meier survival analysis was done looking at transplant-free overall survival for patients diagnosed at our institution. The log-rank test was done to compare survival between patients with and without prior access to primary care, and between patients with and without insurance at diagnosis.

**Results:**

Overall 5- and 10-year transplant-free overall survival was 91 % (95 % CI, 83-100 %) and 75 % (95 % CI, 50-99 %), respectively. Patients with primary care prior to diagnosis had significantly better transplant-free overall survival than those without (log rank test *p* = 0.019). Patients with primary care also had better clinical markers at diagnosis. Having insurance at diagnosis was not associated with better outcomes.

**Conclusions:**

Outcomes of autoimmune hepatitis are poor in our setting but access to primary care prior to diagnosis was associated with better outcomes. This is likely due to the important role that primary care plays in detecting disease and initiating treatment earlier. With the expansion of access to healthcare that the Affordable Care Act provides, future patients are likely to do better with even rare diseases like autoimmune hepatitis.

## Background

Autoimmune hepatitis (AIH) is an inflammatory condition of the liver that can cause chronic hepatitis and cirrhosis. Without treatment, 10-year survival rates are less than 30 %, while with treatment, the 10-year transplant-free survival rate ranges from 85-95 % [1–4]. This data, however, describes mostly non-Hispanic white patients with insurance, in quaternary care settings. Data on outcomes in patients of other ethnicities, especially Hispanics, and in safety-net or low socioeconomic settings are lacking.

In this report, we describe our experience with autoimmune hepatitis in a large urban public hospital in Los Angeles, California that serves a mostly indigent, Hispanic population with higher rates of poverty and lower access to healthcare compared to the county average. At diagnosis with autoimmune hepatitis, some patients are already established within the county primary care network while others are presenting to the county health care system for the first time. Likewise, some patients already have access to some form of insurance (e.g. Medicaid, county-based funding) while others do not. Our aim was to see if having primary care or insurance at diagnosis was associated with better outcomes.

## Methods

This study was conducted in a 350-bed county-run hospital that serves a mostly indigent population, a substantial portion of which cannot qualify for liver transplant because of undocumented immigration status. The hospital’s inpatient discharges are 50 % covered by Medicaid and 20 % covered by county funding. As a surrogate indicator of patients’ socioeconomic background, we used patients’ home zip codes to determine, by geographical area, median household income, proportion living under the federal poverty line [5], and whether the patient lived in an area designated as a “Medically Underserved Area” (MUA) or “Health Professional Shortage Area” (HPSA) [6]. These were included in patients’ baseline characteristic data.

We performed an anonymous retrospective review of patients newly diagnosed with autoimmune hepatitis and managed at our institution between 2001 and 2013. This study was exempt from obtaining consent to access patient data. These patients presented to the gastroenterology service either through referrals from primary care physicians within our county network of clinics or by presenting to our emergency room and being admitted. We queried “Autoimmune Hepatitis” in our pathology department’s database and “Autoimmune Hepatitis” in our hospital’s registry of patients’ ICD-9 diagnoses. Of the patients found in this query, we included patients whose clinical documentation indicated a gastroenterologist’s diagnosis of autoimmune hepatitis supported by clinical findings (liver biopsy, biochemical data, history, etc.) consistent with the revised International Autoimmune Hepatitis Group (IAHG) clinical criteria [7]. Liver biopsies were evaluated by pathologists at our facility and findings of lymphoplasmacytic infiltration or interface hepatitis were considered consistent with AIH. Patients were diagnosed with cirrhosis based on findings on liver biopsy. Exclusion criteria included lack of liver biopsy, age less than 18, diagnosis at another facility, incomplete records, and alternative/concurrent diagnosis of liver disease (e.g. viral hepatitis or overlap syndrome).

Demographic data was obtained from the central hospital database. Ethnicities were self-reported: non-Hispanic white (i.e. Caucasian), Hispanics, African Americans and Asian/Pacific-Islander (i.e. Asians).

Our institution’s gastroenterologists made treatment decisions, including those regarding medication dosing. Patients with AIH were initially treated with prednisone at an oral dose of 30–60 mg daily or methylprednisolone at an intravenous dose of 60 mg daily. Azathioprine or 6-mercaptopurine was either added at a dose of 50–75 mg to prednisone initially or if biochemical and clinical responses were noted within 4–8 weeks. Once response was noted, prednisone was gradually reduced and tapered off within 1 year. Moreover, liver function tests were performed every 1- to 3- months on maintenance doses of prednisone and/or an immunomodulating agent. Response and remission were defined per clinicians’ subjective assessment of biochemical and clinical data.

We recorded two sets of outcomes: 1) a combined endpoint of death and/or liver transplant and 2) a combined endpoint of death, liver transplant, or being lost to follow-up. Follow-up time was defined as time from presentation to a primary endpoint or time of last follow-up. Being lost to follow up was defined as the absence of a clinic visit at any of our hospital’s departments or any laboratory evaluation for 18 months or more after their last clinic visit or laboratory study.

We compared patients with and without primary care at diagnosis for differences in outcomes. We also compared patients with and without insurance at diagnosis for differences in outcomes. Patients were included in the group with primary care if they had at least six months of Internal Medicine or Family Medicine visits at a Los Angeles Department of Health Services facility prior to diagnosis with AIH. Patients were included in the group with insurance if they had Medicaid, Medicare, private insurance, or a form of Los Angeles County funding (for patients who do not qualify for Medicaid) at the time of diagnosis.

All statistical analyses were performed using the SPSS 18 (SPSS Inc. Released 2009. PASW Statistics for Macintosh, Version 18.0. Chicago: SPSS Inc). The chi-squared test and Fisher’s exact test were used to make comparisons of categorical data, the student’s *t*-test was used to compare parametric continuous data, and the Mann–Whitney *U* test was used to make comparisons of nonparametric continuous data. Baseline characteristic data was compared between groups stratified by having primary care and by having insurance. Log-rank testing was done to compare the survival of two groups. Overall transplant-free survival and overall transplant-and-lost-to-follow-up-free survival was analyzed using the Kaplan-Meier method and the log-rank test was used to compare survival between groups. A P-value ≤0.05 was considered statistically significant.

### Ethical approval and consent

This study was deemed exempted by the John Wolff IRB of the Los Angeles Biomedical Research Institute at Harbor-UCLA Medical Center.

### Availability of supporting data

The data set supporting the results of this article is available in the Labarchives respository [http://www.labarchives.com] associated with the DOI 10.6070/H4G44N84.

## Results

One hundred and fifty patients were identified. All patients had an assessment of clinical data included symptoms at presentation, concurrent liver diseases, laboratory data (e.g. liver function panel, ANA, smooth muscle antibody) and cirrhosis. Model for End-Stage Liver Disease (MELD) scores were calculated for patients with cirrhosis using clinical data from presentation to represent severity of liver disease at that time [8]. Sixty-seven were excluded for the following reasons: 15 were excluded because they lacked a liver biopsy, 5 were diagnosed at another facility, 2 had incomplete records, 4 had primary biliary cholangitis (PBC), 1 had alcoholic hepatitis, 1 had an alternative unknown diagnosis, 22 had AIH with overlap syndrome (21 with PBC and 1 with primary sclerosing cholangitis), and 7 had viral hepatitis. Eighty-three patients met IAHG criteria for autoimmune hepatitis and were included for final analysis.

**Fig. 1:**
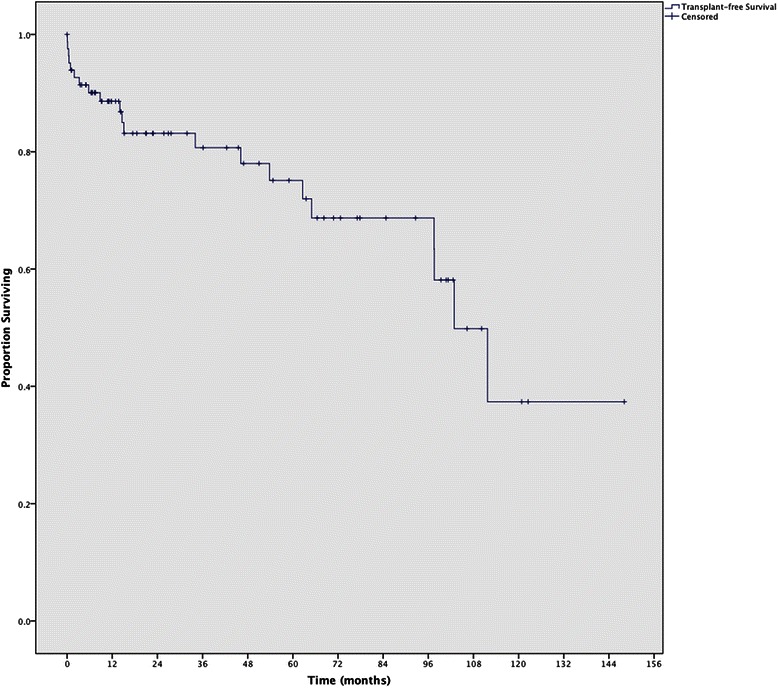
Overall survival

Baseline characteristic data for the entire cohort are described in Table 1. 84 % were female and 84 % were Hispanic, 10 % Asian, and 6 % White. The median estimated household income was $43,364 in our patients’ home zip codes, compared to a county average of $56,241; the median proportion of the population living under the federal poverty line was 22 % in our patients’ zip codes, compared to the county rate of 17 % [9]. 51 % of our patients were from areas designated MUA/HPSA. 39 % presented with cirrhosis, and 7 % presented with decompensated cirrhosis. There were no asymptomatic patients. 49 % scored a definite diagnosis and 51 % scored a probable diagnosis. Sixty-seven patients were treated. Median follow-up time was 21 months. There were 18 deaths and 3 transplants at the time of data collection. 31 % were lost to follow-up. Overall 5- and 10-year transplant-free overall survival was 91 % (95 % CI, 83-100 %) and 75 % (95 % CI, 50-99 %), respectively (Fig. 1). Because a large portion of the cohort was lost to follow-up, we did an additional two-tailed sensitivity analysis for patients lost to follow-up, in which the upper limit assumed patients lost to follow-up survived without transplant and the lower limit assumed patients lost to follow-up died or had a liver transplant; in this case, 5- and 10- year survival rates for the lower limit were 50 % (95 % CI, 62-37 %) and 18 % (95 % CI, 32-4 %), respectively, and for the upper limit, 85 % (95 % CI, 93-77 %) and 81 % (95 % CI, 91-73 %), respectively.

**Table 1:** Baseline characteristics

	Full cohort	Without PCP	With PCP	*p*
n	83	57	26	
Female (%)	70 (84)	47	23	0.746
Ethnicity (Hispanic:Asian:White) (%)	70:8:5 (84:10:6)	46:7:1	24:1:1	
Median age at presentation (yr) (IQR)	48 (41–57)	48 (40–59)	46 (41–55)	0.776
Funding^a^ (%)	48 (58)	29 (51)	19 (73)	0.057
Medicare	4	3	1	
Medicaid	22	15	7	
County Funding	22	9	13	
MUA/HPSA^b^ (%)	42 (51)	31	11	0.307
Poverty Rate of Zip Code (IQR)	22.1 (15.6-26.2)	24.5 (15.6-29)	19.4 (12.9- 23.8)	0.267
Median Income of Zip Code (IQR)	43364 (38903–55784)	43364 (35836–53738)	45556 (42712–57586)	0.160
Median follow-up (mo) (IQR)	21 (7.5-65.7)	15 (6.5-61)	44 (11–89)	0.209
Lost to follow-up (%)	26 (31)	23 (40)	3 (12)	0.010
Presented with Decompensation (%)	6 (7)	6 (11)	0 (0)	0.170
Acute Disease (Symptoms < 2 week) (%)	37 (45)	26 (46)	11 (42)	0.779
Cirrhosis at presentation (%)	32 (39)	26 (46)	6 (23)	0.05
Any decompensation (%)	26 (31)	21 (37)	5 (19)	0.109
ANA >1:40 (%)	59 (71)	37 (65)	22 (85)	0.075
SMA (%)	58 (70)	38 (67)	20 (77)	0.557
LKM (60 checked) (%)	4 (5)	3 (5)	1 (4)	0.762
ANA or SMA (%)	75 (90)	50 (88)	25 (96)	0.425
Other autoimmune disease (e.g. lupus, rheumatoid arthritis, etc.) (%)	23 (28)	12 (21)	11 (42)	0.048
AST (IQR)	429 (157–1248)	487 (136–1329)	418 (168–1074)	0.871
ALT (IQR)	657 (152–1450)	700 (179–1564)	427 (143–942)	0.525
Bilirubin (IQR)	5.2 (1.5-21.2)	11.9 (2.1-22.9)	2.1 (0.7-10.3)	0.040
Albumin (IQR)	2.5 (1.8-3.2)	2.4 (1.8-2.8)	3.1 (2.1-3.5)	0.019
INR (IQR)	1.3 (1.1-1.8)	1.4 (1.1-2.0)	1.1 (1.0-1.3)	0.007
Platelets (IQR)	160 (90–120)	146 (84–202)	174 (114–219)	0.009
IgG (IQR)	1830 (1500–2350)	1820 (1475–2530)	1850 (1500–2170)	0.871
MELD Score (in patients with cirrhosis) (IQR)	17.1 (11.5-24.0)	17.9 (13.0-24.5)	13.9 (9.2-19.4)	0.244
Flu-like symptoms (%)	22 (27)	15 (26)	7 (27)	0.954
Abdominal pain (%)	36 (43)	24 (42)	12 (46)	0.730
Jaundice (%)	54 (65)	41 (72)	13 (50)	0.052
Pruritis (%)	6 (7)	4 (7)	1 (4)	0.660
GI bleed (%)	6 (7)	6 (11)	0 (0)	0.170
Ascites (%)	23 (28)	20 (35)	3 (11)	0.034
Encephalopathy (%)	10 (12)	8 (14)	2 (8)	0.494

^a^Funding was defined as patient having Medicaid, Medicare, Private Insurance, or a County form of insurance

^b^MUA = Medically Underserved Area, HPSA = Health Professional Shortage Area

**Fig. 2:**
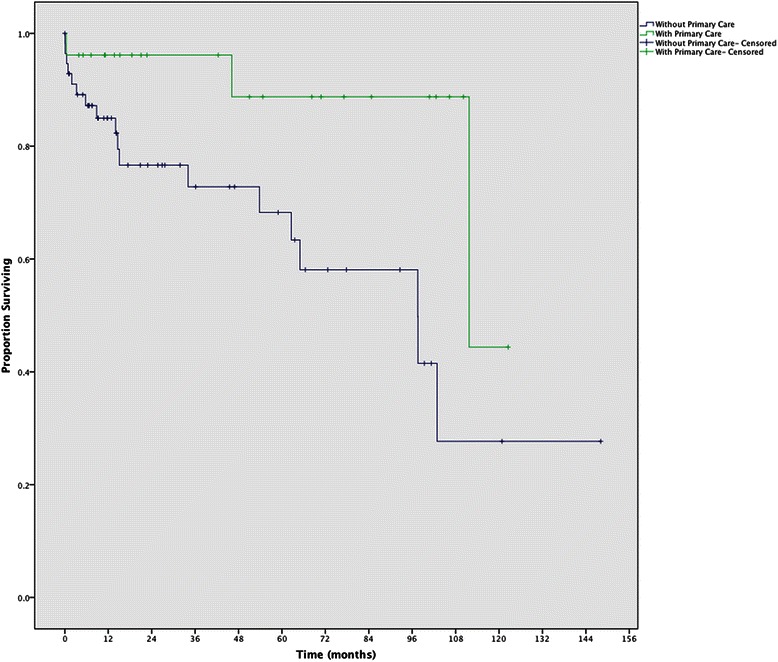
Comparison of survival by primary care access

There was a significant difference in transplant-free survival between patients who had primary care compared to those without (log rank test *p* = 0.019, Fig. 2). 5- and 10- year transplant-free survival rates for patients with primary care were 89 % (95 % CI, 100-73 %) and 45 % (95 % CI, 100-0 %), respectively, and for patients without primary care 5- and 10- year rates were 68 % (95 % CI, 84-53 %) and 28 % (95 % CI, 55-1 %), respectively. There were no significant differences in baseline characteristics or survival between patients with and without insurance at the time of diagnosis.

Patients without prior primary care access at diagnosis were more likely to be lost to follow-up, and had significantly worse markers of liver disease such as cirrhosis on biopsy, platelets, INR, albumin, bilirubin, and ascites (Table 2). Patients without prior primary care access were also much less likely to get treatment.

**Table 2:** Comparison of patients with and without primary care follow-up

	Full cohort	Without PC^a^	With PC^a^	*p*
	N (%)	N (%)	N (%)	
n	83	57	26	
Treated	67 (81)	41 (72)	26 (100)	0.002
Not treated due to decompensation/Childs B or C disease	6	6	0	
Not treated because "No indication" according to clinician	2	2	0	
Not treated because LFTs or liver enzymes either were normal, insignificantly elevated, or resolved on own	5	5	0	
Unclear why not treated	2	2	0	
Patient to follow up with PC physician	1	1	0	
Remission	43 (52)	28 (49)	15 (58)	0.469
Noncompliance	10 (12)	6 (11)	4 (15)	1.000
Steroid median dose	40 (IQR 40–60)	40 (IQR 40–60)	40 (IQR 40–55)	0.535
Median time corticosteroids (months)	12 (14.5)	12 (IQR 5–23)	15 (IQR 12–28)	0.115
Median time to ALT normalization (months)	1.4 (IQR 0.8-3.3)	1.3 (IQR 0.8-3.7)	1.6 (IQR 0.6-2.9)	0.504
Relapses	23 (27)	12 (21)	11 (42)	0.380

^a^PC = Primary Care (Defined as 6 months or greater of Internal Medicine and Family Medicine visit physician follow-up)

## Discussion

Our report shows that in a unique cohort of AIH patients who are predominantly Hispanic treated at an urban safety-net hospital, lack of primary care was associated with worse disease characteristics at diagnosis and worse outcomes. Patients lacking primary care, at diagnosis, had worse rates cirrhosis and ascites, and worse laboratory markers of liver dysfunction (e.g., lower albumin, lower platelets, higher bilirubin). These patients also had higher rates of death and liver transplant.

AIH is a disease that often has a long subclinical course that leads to development of end-stage liver disease. Given this, we hypothesized that patients with regular primary care will likely have their disease diagnosed in earlier stages and have better outcomes than those without primary care. Our results support this supposition. Interestingly, having insurance was not a significant factor associated with better outcomes, suggesting that patients need to actually see their physicians to benefit. In addition, AIH is a disease with good prognosis when treated, but significantly fewer patients lacking primary care in our cohort were treated due to their having Childs B or C cirrhosis or other acute illnesses such as sepsis.

Patients in our cohort had important factors that made them less likely to have access to primary care. They lived in areas with lower incomes and higher rates of poverty and shortages of doctors compared to the county average [9] and they also had much lower rates of insurance coverage compared to the national average [10]. Hispanics, of which our cohort was mostly composed, are disproportionately likely to be of low socioeconomic status, lack access to health care and use safety net hospitals [11].

With the implementation of the Affordable Care Act, we hope to see expanded primary care access for the previously uninsured—particularly for those living in poverty, and for Hispanics. Through many encounters over time, improved management of treatable chronic illnesses with high morbidity and mortality, like AIH, can be achieved. Ensuring good follow up will be essential for producing good outcomes, but in safety net settings, where many patients with poor access to health care are treated, this has been challenging [12]. Active changes are being made in our own institution, such as patient-centered medical homes, electronic specialty consultation from primary care clinics, and a new electronic medical record, with the hope that these improvements may improve this problem. This should help primary care physicians and specialists better coordinate care and be more vigilant against losing patients due to systemic issues.

Our study had some important limitations. Comparisons between ethnicities were not possible because of the cohort’s small size. Given that AIH is a relatively rare disease, large, prospective studies are likely to be difficult, especially in the safety-net setting. Its retrospective nature makes it vulnerable to bias. Endpoints such as remission and relapse were subjectively defined by clinicians and sometimes did not strictly adhere to AASLD-defined criteria, and this increases risk of bias as well.

## Conclusion

In summary, this report shows that access to primary care prior to the diagnosis of AIH in a safety net hospital setting with mostly Hispanic patients is associated with better outcomes, while merely having insurance at presentation is not associated with any difference in outcomes. Patients who lacked primary care access also presented with worse liver disease.

## Abbreviations

AIH: Autoimmune hepatitis
MUA: Medically underserved area
HPSA: Health professional shortage area
IAHG: International Autoimmune Hepatitis Group
MELD: Model for end-stage liver disease
